# The significance of lipid metabolism reprogramming of tumor-associated macrophages in hepatocellular carcinoma

**DOI:** 10.1007/s00262-024-03748-9

**Published:** 2024-07-02

**Authors:** Qingjian Xie, Yuan Zeng, Xiangting Zhang, Fujun Yu

**Affiliations:** 1 Department of Neurology, The First Affiliated Hospital of Wenzhou Medical University, Wenzhou, Zhejiang China; 2 Department of Gastroenterology, The First Affiliated Hospital of Wenzhou Medical University, Wenzhou, Zhejiang China

**Keywords:** Tumor-associated macrophages, Hepatocellular carcinoma, Lipid metabolic reprogramming, Tumor microenvironments

## Abstract

In the intricate landscape of the tumor microenvironment, tumor-associated macrophages (TAMs) emerge as a ubiquitous cellular component that profoundly affects the oncogenic process. The microenvironment of hepatocellular carcinoma (HCC) is characterized by a pronounced infiltration of TAMs, underscoring their pivotal role in modulating the trajectory of the disease. Amidst the evolving therapeutic paradigms for HCC, the strategic reprogramming of metabolic pathways presents a promising avenue for intervention, garnering escalating interest within the scientific community. Previous investigations have predominantly focused on elucidating the mechanisms of metabolic reprogramming in cancer cells without paying sufficient attention to understanding how TAM metabolic reprogramming, particularly lipid metabolism, affects the progression of HCC. In this review article, we intend to elucidate how TAMs exert their regulatory effects via diverse pathways such as E2F1-E2F2-CPT2, LKB1-AMPK, and mTORC1-SREBP, and discuss correlations of TAMs with these processes and the characteristics of relevant pathways in HCC progression by consolidating various studies on TAM lipid uptake, storage, synthesis, and catabolism. It is our hope that our summary could delineate the impact of specific mechanisms underlying TAM lipid metabolic reprogramming on HCC progression and provide useful information for future research on HCC and the development of new treatment strategies.

**Supplementary Information:**

The online version contains supplementary material available at 10.1007/s00262-024-03748-9.

## Introduction

Liver cancer, notably hepatocellular carcinoma (HCC), represents a significant global health burden, with its prevalence projected to escalate [1]. Historically, therapeutic options for HCC have been largely confined to the use of anti-angiogenic tyrosine kinase inhibitors (TKIs) and molecularly targeted agents with anti-proliferative properties, albeit challenged by the emergence of resistance mechanisms within cancer cells. In recent years, the therapeutic approaches have begun shifting toward immunotherapy, with particular attention focusing on immune checkpoint inhibitors (ICIs) [2, 3]. This transition underscores the imperative for a comprehensive understanding about immune cell functionality within the HCC microenvironment.

Over the past two decades, there has been a burgeoning interest in cancer metabolism, with considerable focus on the metabolic reprogramming of cancer cells, including the well-documented "Warburg effect" involving glucose metabolism [4, 5]. In this context, lipid metabolism and reprogramming have received increasing attention. Emerging evidence highlights the pivotal role of aberrant lipid metabolism in cancer cells, which is crucial for their proliferation, metastasis, and invasive behavior [6]. In addition, this phenomenon fosters the establishment of a tumor microenvironment (TME) characterized by elevated lipid concentrations, thereby orchestrating lipid metabolic reprogramming in diverse immune cell subsets within the TME. For instance, fatty acid (FA) uptake and intracellular lipid content were augmented in T regulatory (Treg) cells of melanoma compared to those in other tissues of the same host [7]. Additionally, studies have shown that the metabolic alterations of various lipids, including glycerophospholipids, sphingolipids, and cholesterol, influence processes such as aberrant growth signaling, metastasis, evasion of cell death, and immune suppression in HCC [8]. Given the pivotal role of immune cells in cancer advancement and the evolving therapeutic landscape of HCC, it is necessary to determine whether immune cells undergo lipid metabolic reprogramming within the TME and elucidate the impact of these processes on immune cell functionality and cancer progression [9].

**Fig. 1:**
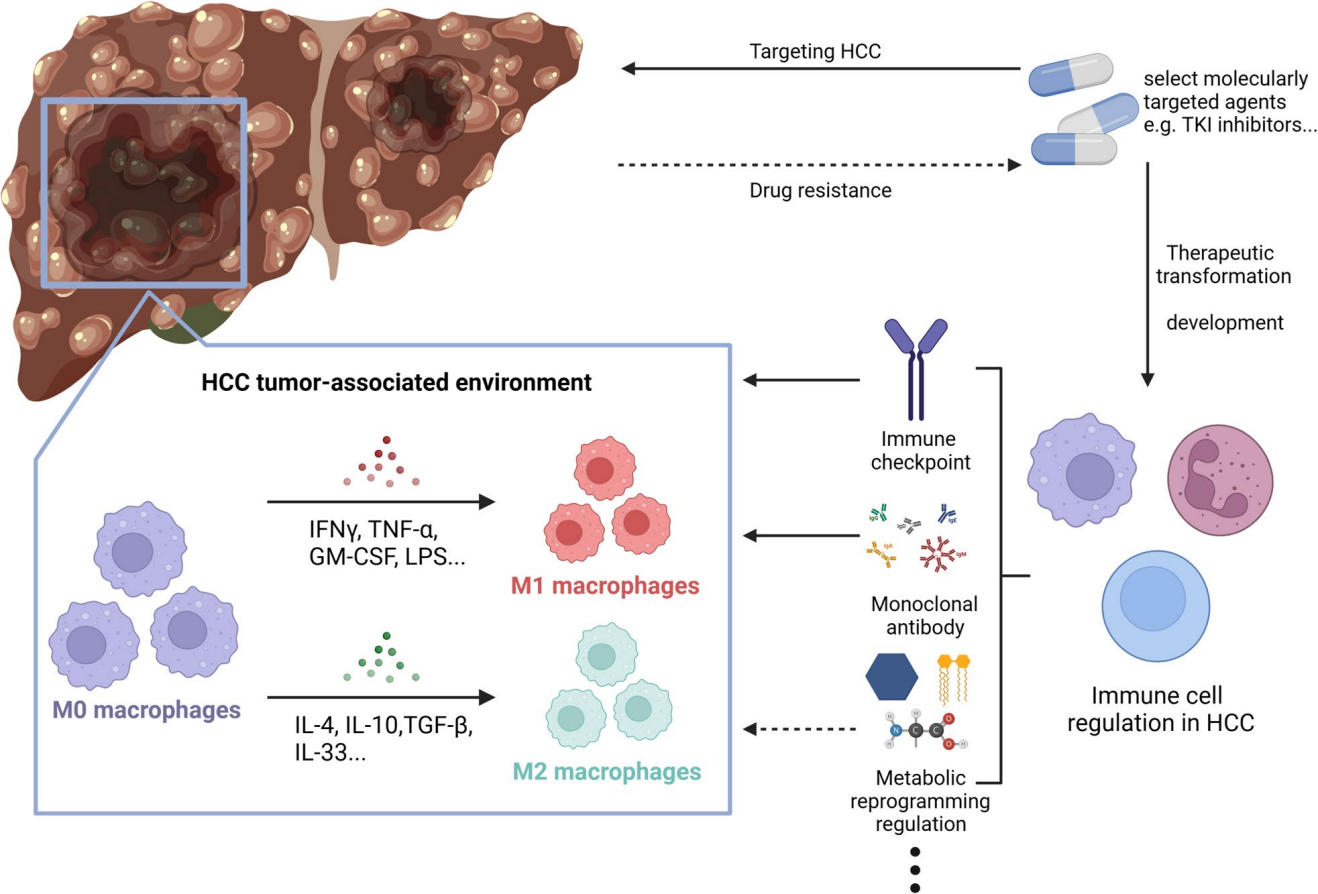
Overview of HCC therapy. (1) As resistance to targeted molecular therapy poses a significant challenge in the treatment of HCC, there is a growing shift toward immunotherapy as a promising alternative. (2) The therapy targeting immune cell regulation encompasses various approaches, including immune checkpoint inhibition, monoclonal antibodies, metabolic modulation, and other methodologies. Among these, research into metabolic regulation, particularly lipid metabolic reprogramming, is still in its nascent stages. (3) Tumor-associated macrophages (TAMs) constitute the primary immune cell population within the HCC microenvironment, exerting significant influence on the progression of HCC. Their polarization is profoundly impacted by various factors, including metabolic cues. Source Created with BioRender.com

TAMs represent the predominant immune cell population within the HCC microenvironment [10]. These TAMs undergo a phenotypic shift toward a tumor-supportive phenotype, which is distinct from macrophages endowed with intrinsic tumor-suppressive capabilities, thereby fostering tumor immunosuppression and trophic functions [11]. This shift is closely linked to adverse prognostic outcomes in HCC patients. Although recent investigations have delineated pivotal pathways governing TAM recruitment, polarization, and metabolism in HCC progression [12], it is still necessary to further clarify TAM lipid metabolic reprogramming in the context of HCC. Previous studies have underscored that macrophages undergo metabolic reprogramming in both innate immunity and adaptive immunity to adapt to aberrant environmental cues such as hypoxia and nutrient fluctuations [13]. Simultaneously, related research indicates that receptor-interacting protein kinase 3 (RIPK3), a core factor of necroptotic signaling, is downregulated in TAMs in HCC. This downregulation promotes FA metabolism and induces M2 polarization within the TME [14]. Motivated by these observations, we postulate that TAMs exert their modulatory effects on cancer progression by undergoing extensive lipid metabolic reprogramming within the HCC microenvironment. Based on this postulation, we synthesize and consolidate the existing literature about TAM-mediated lipid metabolic reprogramming within the TME, then contextualize these findings within the unique milieu of the HCC microenvironment, and finally deliberate on the potential implications of TAM lipid metabolic reprogramming for future therapeutic strategies targeting HCC (Fig. 1).

## Tumor-associated macrophages in HCC

TAMs originate from monocytes infiltrating solid tumor tissues from the peripheral blood and constitute a substantial portion of immune cells in various tumor types. They exhibit functional diversity and are governed by signals from tumor cells, T and B lymphocytes, and stromal cells [15]. Although the typological classification of TAMs has been a subject of debate, the prevailing consensus recognizes two primary phenotypic states: classically activated macrophages (M1) and alternatively activated macrophages (M2) [16, 17]. M1 polarization typically occurs in response to inflammatory cues like interferon-gamma (IFN-γ) and lipopolysaccharide (LPS), culminating in potent tumoricidal effects and promotion of hemorrhagic tissue necrosis via secretion of inflammatory cytokines such as interleukin-12 (IL-12), IL-6, tumor necrosis factor (TNF), as well as reactive nitrogen and oxygen intermediates [9, 15]. Conversely, M2 polarization is induced by anti-inflammatory signals such as IL-4, IL-10, and glucocorticoids, leading to enhanced secretion of anti-inflammatory mediators and facilitating tissue remodeling and immunosuppression by dampening the release of inflammatory factors [9]. Notably, M2 polarization fosters immune evasion and tumor progression by upregulating programmed death-ligand 2 (PD-L2) expression in TAMs via the PD-1 signaling pathway [18]. In addition, there exists a spectrum of TAM phenotypes between the M1 and M2 states within the TME, and these TAM phenotypes are affected by various factors such as nutrient availability, pH, oxygen level, and cytokine milieu, among others [19].

In the TME of HCC, there exhibit certain differences in the types and characteristics of TAMs. As HCC is a highly malignant and heterogeneous tumor, TAMs associated with its function are predominantly of the M2-type. They promote the development of HCC by secreting various cytokines and activating related signaling pathways such as IL-6/JAK/STAT3 and NF-κB pathways [20]. In response to these characteristics of TAMs, multiple studies have sought to treat HCC by modulating the polarization of TAMs. For instance, the combination of arsenic trioxide (ATO) and cryptotanshinone (CTS) was found to induce glycolysis in TAMs by activating the AMPK pathway, thus promoting polarization toward an M1-like macrophage phenotype [21]. Consequently, the distinct implications of different TAM polarization states in HCC progression necessitate a comprehensive exploration to clarify how their lipid metabolic processes modulate TAM polarization.

## Lipid metabolism in TAMs

Lipids play a critical role in the development of TAMs within the HCC microenvironment, primarily derived from the extensive uptake by macrophages themselves and the heightened de novo synthesis of lipids after the reprogramming of lipid metabolism in cancer cells [22, 23]. Conversely, throughout the differentiation of diverse TAM subsets, the processes of lipid metabolism within TAMs, including lipid uptake and storage and synthesis (comprising cholesterol, FA and phospholipids), and oxidative lipid metabolism, undergo corresponding reprogramming, which is believed to be intimately linked with HCC progression and alterations in the microenvironment.

### Lipid transport

Lipid transport encompasses several processes including uptake, excretion, transport, and utilization. It plays a critical role in providing synthetic metabolic precursors for HCC and TAMs by participating in signal regulation and other functions. Raw materials for lipid metabolism are generally derived from both exogenous uptake and de novo synthesis, among which FA is the primary form of lipids exogenously absorbed and used for energy production. Macrophages have been shown to endocytose various low-density lipoproteins such as acLDL and ox-LDL through scavenger receptor (SR)-mediated mechanisms [24]. Within the TME, animal experimental evidence has demonstrated that TAMs increase FA uptake by upregulating the expression of the FA translocase CD36, which functions as a type of scavenger receptor [23]. Additionally, single-cell RNA sequencing of phase III human HCC samples has revealed an overexpression of FA-binding protein 1 (FABP1) in TAMs. This overexpression works in conjunction with peroxisome proliferator-activated receptor γ (PPARG)/CD36 to facilitate the transport and oxidation of FA [25]. Concurrently, fat uptake by TAMs is influenced by IL-25, knowing that high IL-25 expression in HCC significantly enhances lipid uptake by macrophages [26, 27]. However, this phenomenon is not uniform across all TAMs. For instance, a previous mouse experiment reported that FA and triglyceride (TG) uptake was enhanced in IL-4-activated M2-like TAMs, whereas FA uptake was inhibited in LPS + IFNg-activated M1-like TAMs [9].

Previous studies have extensively analyzed the gene expression profiles of HCC and observed increased expression of CD36 and caveolin-1 (CAV1) in HCC cells, akin to TAMs, resulting in heightened FA uptake. Moreover, biosynthetic genes for FA were found to be generally upregulated in most HCC tissues compared to normal non-HCC tissues, indicating increased rates of de novo lipid synthesis [28, 29]. Furthermore, adipose triglyceride lipase (ATGL) was found to be highly expressed in human HCC tissues, while FA-related oxidases such as carnitine palmitoyl transferase 1A (CPT1A) and acyl-coenzyme A oxidase 1 (ACOX1) were found to be downregulated, leading to increased breakdown of stored lipids in HCC cells and elevated levels of diglycerides (DAG) and free FA (FFA) [30, 31]. Given the evidence that activation of the E2F1-E2F2-CPT2 axis in nonalcoholic fatty liver disease (NAFLD)-related HCC promotes a lipid-rich environment conducive to HCC progression, alongside the observed stimulation of increased lipogenesis in various cell lines under hypoxic conditions within the HCC microenvironment [32, 33], we hypothesize that the HCC microenvironment as a whole remains highly enriched in lipids, notwithstanding the state of heightened lipid uptake, synthesis, and metabolism in HCC cells. In summary, given the combined effects of lipid uptake by M1 and M2 TAMs, it is reasonable to speculate that the lipid uptake reprogramming mechanism of M2 TAMs may be better adapted to the lipid-rich microenvironment of HCC.

**Fig. 2:**
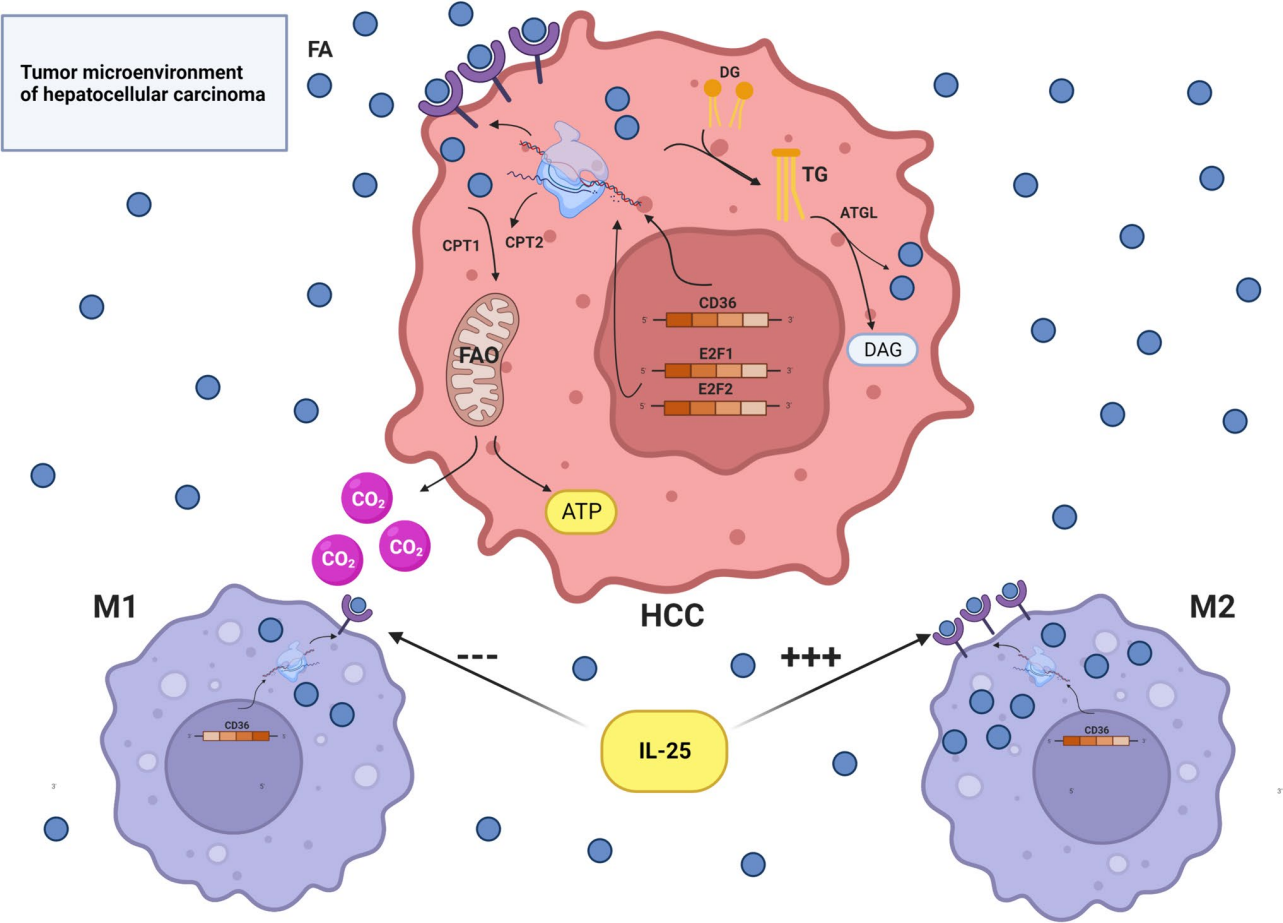
Lipid transport in HCC TME involves several key mechanisms: (1) Fatty acids (FAs) are extensively taken up due to heightened expression of CD36 in hepatocellular carcinoma cells, as well as in M1 and M2 macrophages. (2) The expression of CD36 in TAMs is regulated by IL-25, with decreased levels in M1 macrophages and elevated levels in M2 macrophages. (3) FAs-related oxidases like carnitine palmitoyl transferase (CP) are downregulated, leading to increased levels of breakdown products such as diglycerides (DAG) and free fatty acids (FFA) in hepatocellular carcinoma cells. (4) The E2F1-E2F2-CPT2 axis is implicated in creating a lipid-rich environment conducive to HCC progression. These mechanisms collectively contribute to the enhanced lipid uptake observed in the HCC TME, facilitating tumor progression and metabolic adaptation. Source Created with BioRender.com

However, considering that nonalcoholic fatty liver disease (NAFLD) is recognized as one of the independent risk factors for HCC [33], it is reasonable to infer that hepatic lipid enrichment plays a pivotal role in HCC progression [33, 34]. Although M2 TAMs typically exhibit tumor-promoting and immune evasion effects in most cancers, the regulation of high-lipid uptake in the HCC microenvironment may disrupt this lipid-rich milieu. Notably, a study has demonstrated that inhibiting hepatocyte uptake of exogenous lipids by targeting lipid lipase (LPL) transcription through ZHX2 can attenuate HCC proliferation [35]. Hence, the disruption of the high-lipid TME may represent a viable therapeutic approach for HCC treatment. Nevertheless, relevant investigations have revealed that macrophages can facilitate IL-4-mediated M2-type TAM reprogramming through processes like cholesterol efflux and lipid raft consumption under the influence of ovarian cancer cells, thereby promoting cancer progression [11]. This phenomenon not only implicates the role of M2 TAMs in tumor progression but also suggests that the lipids that they release may be absorbed and utilized by cancer cells. Furthermore, cancer cells often upregulate LDL receptors (LDLR) and enhance cholesterol uptake as an alternative strategy for proliferation [36]. Meanwhile, several studies have demonstrated that exosomes produced by TAMs are transferred to HCC cells, eliciting various effects such as promoting or inhibiting HCC cell proliferation and enhancing metastasis [37–39]. These exosomes are rich in various lipids, including phospholipids, sphingolipids, cholesterol, triglycerides, and fatty acids [40]. While existing research has not directly proved that these lipids are involved in promoting HCC proliferation, based on the evidence currently available, we can hypothesize that one or more lipids released from TAMs, either through excretion or post-apoptotic release of FA and other lipids, could provide an energy source for the tumor, though this hypothesis requires further experimental validation. In addition, exploring approaches that can enhance TAM lipid uptake on the one hand and inhibit TAM lipid release on the other hand to attenuate HCC progression, along with strategies to modulate M2-type polarization and augment M1-type polarization, could represent promising directions for future research (Fig. 2).

### Lipid storage

Similar with other cells, TAMs typically store excess FA intracellularly in the form of lipid droplets (LD) by synthesizing TG with glycerol. Consequently, TAMs, along with HCC cells, upregulate the expression of FA-binding protein (FABP) in response to FA stimulation, thereby regulating lipid uptake and storage within the high-fat HCC microenvironment [41]. Conversely, recent studies have highlighted lipid accumulation as a critical hallmark of HCC, with high LD levels considered indicative of cancer invasion across various tumor types [42]. So we speculate that lipid accumulation in TAMs may function as a nutritional reserve for HCC progression and play a tumor-promoting role.

Furthermore, the aforementioned study revealed that activation of AMP-dependent protein kinase (AMPK) contributed to the attenuation of antecedent lipid accumulation in the liver induced by high glucose or high-fat diets. Liver kinase B1 (LKB1) is identified as an upstream pathway of AMPK, which activates the LKB1-AMPK signaling cascade to impede the progression of liver disease [42]. Concurrently, the regulation of lipid accumulation in hepatocytes by hepatic macrophages (Kupffer cells) is primarily mediated by the P2X7 receptor-NLRP3 inflammasome pathway, leading to activation of the LKB1/AMPK signaling cascade [43, 44]. However, no direct evidence is available to support the role of Kupffer cells in regulating lipid accumulation through this pathway. Currently, research relevant to this topic is primarily concentrated in the cardiovascular system. Relevant research has demonstrated that reduced macrophage LKB1 activation induced by ox-LDL in the cardiovascular system promotes lipid uptake, storage, and the formation of foam cells in macrophages [45]. Therefore, Kupffer cells, which may function as TAMs in the HCC microenvironment, could experience LKB1 deficiency due to various factors. This deficiency might not only promote lipid accumulation in HCC cells via this pathway but could also lead to excessive lipid accumulation within the cells themselves. This signaling axis may represent a potential target for modulating lipid metabolism and inhibiting HCC progression, which underscores the importance of exploring therapeutic strategies that intervene in lipid accumulation pathways to counteract tumor-promoting processes in the liver microenvironment. It is also noteworthy that studies on the impact of OS-LDL on macrophage function in the cardiovascular system have highlighted the cytotoxic effects of excessive OS-LDL and free cholesterol (FC) accumulation in macrophages, leading to rapid apoptosis [46]. Therefore, it warrants further investigation to ascertain whether OS-LDL and its induced lipid uptake also exert cytotoxic effects in Kupffer cells and their potential implications for HCC progression.

Farnesoid X receptor (FXR), a multifunctional transcription factor, plays a crucial role in preventing liver cancer development. Studies have revealed that FXR inhibits the growth of HCC cells by upregulating microRNA-122 (miR-122) [47]. MiR-122, the most abundant and liver-specific miRNA, is known to be stimulated by elevated levels of FFA in the body. This upregulation of miR-122 can mitigate TG accumulation in both liver and peripheral tissues [48]. Investigations above further indicate that administration of miR-122 inhibitors and FFA inducers such as CL316243 to mice results in TG accumulation in liver and muscle tissues, accompanied by a reduction in β-oxidation rates. This underscores the widespread physiological effects of miR-122 and its potential relevance to macrophages. Besides, lipid-exposed hepatocytes release miR-122, which can be absorbed by liver tissue macrophages, activating them to produce inflammatory cytokines. Additionally, miR-122 promotes M1 polarization by modulating PPARδ and NF-κB signaling pathways, which is conducive to forming an anti-HCC environment [49, 50]. Therefore, the above evidence indicates that high-level absorption of lipids by TAMs and their high-level storage within cells do not seem to benefit the formation of a high FFA environment to activate FXR; rather, they may lead to HCC progression. But whether miR-122 truly interacts with the lipid storage in TAMs within the liver cancer TME deserves further research, knowing that it could provide insights into the complex interplay between miRNA regulation, lipid metabolism, and macrophage function in the context of liver cancer.

In addition to summarizing the reprogramming of various lipid uptake and storage pathways in TAMs within the HCC microenvironment, it is crucial to acknowledge the broader alterations resulting from their regulation. Animal experiments have revealed that CD36 is upregulated in metastasis-associated macrophages (MAM) within the HCC microenvironment, promoting the development of hepatic metastasis and fostering a heightened infiltration of M2-like MAM, thereby creating a highly immunosuppressed TME. Concurrently, elevated levels of IL-25 in HCC stimulate the polarization of M2-like TAMs [26, 51]. Furthermore, while LKB1 is implicated in the regulation of lipid metabolism, its role extends to the modulation of glucose, glutamine, and serine metabolism, which significantly impact cellular processes [42].

Conversely, various metabolites of glucose, glutamine, and serine can reciprocally influence lipid synthesis. For instance, glutamine can be converted to α-ketoglutarate via alanine serine cysteine preference transport protein 2 (ASCT2), which is highly expressed in HCC, and subsequently enters cellular mitochondria. Here, α-ketoglutarate can be converted to citric acid and transported out of mitochondria through reductive carboxylation. The breakdown of citric acid in the cytoplasm yields acetyl coenzyme A, which is a raw material for de novo FA synthesis [52, 53]. Thus, the inhibition of CD36 expression and the modulation of the LKB1 signaling pathway emerge as potential strategies for future HCC treatment. Understanding the intricate interplay between lipid metabolism and broader cellular metabolic processes holds promise for developing effective therapeutic interventions for HCC.

It is noteworthy that autophagy seems to play a significant role in regulating lipid uptake and storage in TAMs. As a catabolic process enabling cells to degrade intracellular components and support survival during stressful conditions, autophagy provides substrates for various aspects of cellular metabolism, including lipids [54]. In the liver, hepatocytes utilize autophagy to supply diverse energy sources, while non-parenchymal cells such as macrophages rely on autophagy to maintain cellular homeostasis and fuel their activation [55]. Studies have indicated that defective autophagy can promote the progression of HCC [56], likely through mechanisms related to the accumulation of intracellular storage lipids, such as LD. The progression of HCC is intricately linked to lipid accumulation, and cellular components like LD are eliminated via the autophagic pathway [22, 57]. Furthermore, inhibition of autophagy has been shown to increase TG and LD accumulation in liver cells following lipid stimulation [58]. However, the specific mechanisms underlying how autophagy regulates lipid metabolism in TAMs and its impact on HCC progression require further investigation. Understanding these processes could offer valuable insights into the role of autophagy in tumor metabolism and provide potential therapeutic targets for HCC treatment.

### Lipid synthesis

TAMs, akin to other cells, engage in FA synthesis through de novo pathways by utilizing them as building blocks to synthesize various lipids tailored to meet their metabolic demands. However, their lipid synthesis process undergoes alterations in response to signals from stromal and cancer cells within the TME.

Human cell culture experiments, particularly those involving TAM induction by non-myeloid thyroid cancer (TC) and neuroblastoma (NB) groups, have shed light on these dynamics. Transcriptomic and metabolomic analyses have revealed a robust upregulation of lipid biosynthetic pathways in TAMs, as evidenced by an increase in total lipid content within TAMs [59]. As with TC and NB, HCC is also a malignant tumor characterized by high TAM infiltration, suggesting that TAM in these tumors may also exhibit excessive activation of lipid synthesis. The latest research reveals that the overexpression of transmembrane protein 147 (TMEM147) in HCC cells increases lipid metabolism and FA content in macrophages by upregulating the expression of 27-hydroxycholesterol (27HC) derived from HCC cells [60]. The aforementioned studies also indicate that increased lipid synthesis metabolism in TAMs can promote M2 polarization through peroxisome proliferator-activated receptor γ signaling, thereby facilitating the invasion and migration of HCC cells. Therefore, the impact of lipid synthesis reprogramming in TAMs on HCC warrants our attention. Here, we explore several pathways that potentially influence TAM lipid synthesis within the HCC microenvironment.

#### LKB1

As previously mentioned, LKB1 may indeed play a crucial role in regulating TAM accumulation of lipid stores. However, it is important to note that LKB1 also holds a significant position in modulating lipid synthesis pathways in TAMs.

From the upstream pathway perspective of LKB1, although there are currently no direct studies on the regulation of lipid synthesis in TAMs in the HCC environment, relevant studies on the reprogramming of macrophage lipid synthesis can provide insight. Liraglutide (LRG), a glucagon-like peptide 1 analog (GLP1A), increases the phosphorylation of LKB1 by promoting the secretion of FGF21, thereby inhibiting TG synthesis in macrophages [61]. The E3 ubiquitin ligase Itchy E3 ubiquitin-protein ligase (ITCH) can promote LDL uptake and lipid accumulation in macrophages by mediating the ubiquitination and degradation of LKB1 [62].

From the downstream pathway perspective of LKB1, acetyl coenzyme A carboxylase (ACC) stands out as one of the pivotal rate-limiting enzymes governing FA de novo synthesis. Its role is to catalyze the carboxylation of acetyl coenzyme A and yield malonyl coenzyme A, which serves as the primary substrate for FA synthesis [63]. Typically, ACC functions as a downstream target within the LKB1-AMPK signaling pathway. Upon pathway activation, ACC undergoes phosphorylation, resulting in the inhibition of its enzymatic activity [64]. However, an intriguing finding from a related study showcased that the liver-specific inhibitor ND-654 effectively retarded HCC progression by impeding lipid synthesis through ACC inhibition [65]. This underscores the significance of ACC in driving HCC pathogenesis and positions it as a promising target for therapeutic intervention in HCC management.

Indeed, recent studies have unveiled that the paradoxical presence of LKB1 overexpression in advanced HCC is correlated with unfavorable survival outcomes [66]. This observation might be attributed to the prolonged metabolic stress endured by HCC cells within their microenvironment. Under such conditions, activation of the LKB1-AMPK pathway confers a survival advantage to cells by orchestrating the stimulation of catabolic pathways and inhibition of anabolic pathways. Consequently, HCC can sustain proliferation even amidst high anabolic conditions. Therefore, the expression levels of LKB1 may vary across different compartments and stages of HCC development. In scenarios where HCC cells dominate the microenvironment, heightened expression of LKB1 becomes imperative to maintain a survival advantage, albeit at the expense of diminished lipid synthesis. This necessitates the uptake of external lipids to fulfill the growth demands. Based on the analysis of "lipid transport" and "lipid storage" in the preceding text, TAMs, as one of the highly infiltrating immune cells in the TME, can theoretically serve as a source of lipids synthesized for liver cancer cells. Previous findings have demonstrated excessive lipid accumulation in LKB1-deficient immune cells such as dendritic cells (DCs) and Tregs [67, 68]. Hence, it is plausible to hypothesize that deficiency or loss of LKB1 in TAMs may lead to hyperactive lipid synthesis, thereby supplying lipids to HCC cells and expediting HCC progression. Consequently, further investigations elucidating the expression patterns of LKB1 during HCC progression across diverse cell populations and their corresponding metabolic reprogramming effects could unveil novel therapeutic targets for HCC treatment.

#### SREBP

Sterol regulatory element-binding protein (SREBP) emerges as a pivotal nuclear transcription factor governing lipid metabolism in eukaryotic cells [69]. Renowned for its central role in cancer cell proliferation [22], SREBP also assumes critical regulatory functions in the reprogramming of lipid metabolism within TAMs [70]. SREBP activation is intricately regulated and modulated by multiple pathways.

The mechanistic target of rapamycin (mTOR) functions as the catalytic subunit of two distinct complexes, namely mTORC1 and mTORC2, which serve as central metabolic regulators in both immunity and AMPK signaling pathways [42]. Recent studies have shed light on the multifaceted roles of mTOR complexes, showing that mTORC1 activation is correlated with enhanced adipogenesis and concurrently inhibits lipolysis and impedes FA oxidation (FAO). Conversely, mTORC2 activation suppresses the conversion of glucose to lipids, although its involvement in positively regulating the FA de novo synthesis pathway has been elucidated in other investigations [71]. Several studies have elucidated the regulatory role of mTORC1 in lipid metabolism, particularly through the mediation of the transcription factor SREBP. Notably, mTORC1 modulates the expression of key lipogenic enzymes such as ACC, FASN, and SCD1 via SREBP [72–74]. SREBP exists in multiple isoforms, with SREBP-1 primarily governing the expression of enzymes involved in FA synthesis, while SREBP-2 predominantly regulates enzymes in the mevalonate pathway [75]. In the context of immune cells, mTORC1 plays a pivotal role in enhancing lipid synthesis by promoting SREBP activation through various signaling proteins including S6K1, CREB, and Lipin1. For instance, during the clonal expansion of effector cells, activated CD8 + T cells necessitate SREBP activation [22, 71, 76]. Although heightened SREBP activation has been observed in hepatocytes during HCC progression [77], the activation status of SREBP in TAMs in HCC remains unexplored. Given SREBP's influence on diverse lipid metabolic pathways, further investigation is warranted to conclusively elucidate its varied effects on TAMs in HCC.

Cholesterol metabolism is a crucial lipid metabolic process in the progression of HCC. Studies have demonstrated that increased cholesterol biosynthesis due to p90 ribosomal S6 kinase 2 (RSK2)-inactivating mutations can enhance the sensitivity of HCC to sorafenib treatment [78]. Additionally, research suggests that cholesterol synthesis can influence the progression of HCC by regulating Yes-associated protein (YAP) and PDZ-binding motif-containing protein (TAZ) functions [79]. However, the impact of cholesterol on immune cells in the TME of HCC should not be overlooked. Research indicates that cholesterol accumulation in NK cells can activate their effector capacity against HCC cells [80]. Cholesterol plays a multifaceted role in macrophages, serving not only as a structural component of cell membranes but also affecting various cellular pathways including respiration and metabolism. Intracellular cholesterol levels are tightly regulated through feedback mechanisms involving the SREBP2 pathway for synthesis and the LDLR gene transcription pathway for uptake [81, 82], as excessive intracellular cholesterol accumulation can produce cytotoxic effects by impairing cell membrane fluidity and membrane protein function, as well as mitochondrial respiration and membrane polarization in macrophages [83]. It has been shown that LPS-activated macrophages can maintain an inhibitory effect on SREBP2 activation and cholesterol synthesis by upregulating cholesterol 25-hydroxylase (Ch25h) [83]. It has also been shown that the SREBP cleavage-associated protein (SCAP) is involved in modulating cholesterol levels by promoting SREBP-1a activation, consequently inhibiting polarization toward the M1-like TAM phenotype [84]. All these findings indicate that cholesterol synthesis plays a crucial role in TAM metabolic reprogramming, potentially influencing HCC progression by modulating the formation of M1-like TAM. Future studies should further investigate the intricate interplay between cholesterol metabolism and TAM polarization in HCC to uncover novel therapeutic avenues for HCC treatment.

FAs, integral to metabolic lipid regulation, can be modulated through the SREBP1 pathway in response to specific stimuli. A pertinent study reported that normal macrophages exhibited increased de novo FA synthesis under LPS stimulation, while SREBP-1a-deficient (SREBP-1aDF) macrophages did not demonstrate lipid accumulation [85]. Knowing that LPS-activated macrophages tend to polarize into M1-like TAM in the HCC microenvironment, we postulate that these cells may sustain cellular function stability by activating the SREBP2 pathway to prevent intracellular cholesterol accumulation. Concurrently, they could employ SREBP-1a activation in a pro-inflammatory manner to combat HCC cells. Existing research has indeed demonstrated that the lipid burden of macrophages in the TME is associated with their anti-tumor and pro-inflammatory capabilities [86]. Therefore, augmented lipid synthesis in TAMs could serve as an energy source for anti-tumor activity and potentially impede HCC progression. Consequently, targeting SREBP activation and preventing SREBP deficiency to harness the full potential of M1-like TAMs hold promise as an immune-based therapeutic approach for future HCC treatment.

The Hippo signaling pathway is a recently identified tumor suppressor pathway, which imposes significant impact on HCC progression primarily by inhibiting HCC cell proliferation and suppressing the transcriptional activity of YAP/TAZ through two Hippo kinases, Mst1 and Mst2 [87]. YAP/TAZ, situated downstream in this pathway, also plays a pivotal role in the reprogramming of lipid metabolism. Experimental evidence indicates that YAP/TAZ, the target of the Hippo signaling pathway, stimulates de novo lipid production by activating the mechanistic target of mTORC1 signaling and inducing downstream activation of SREBP [88].

Related studies have demonstrated that the expression of inflammatory factors such as IL-1β, Mcp1, and IL-6 was elevated while the expression of macrophage inflammatory protein-1β (MIP-1β) and similar immune cell-encoded chemoattractants was decreased in the liver of Mst1 and Mst2 deficient mice [89]. This dysregulation contributes to liver overgrowth and the formation of tumor nodules [87]. It is hypothesized that inflammatory factors like IL-1β, Mcp1, and IL-6 serve as chemotactic and proliferative signals for macrophages, biasing the process of macrophage polarization toward the M1-type in response to inflammation, thereby accelerating the formation of a highly infiltrated macrophage environment. Conversely, macrophages themselves exhibit low expression of MIP-1β, suggesting an overall bias toward M2-type polarization in the TME after establishing a high macrophage infiltration environment, which favors an immunosuppressive environment to inhibit inflammatory tumor eradication and promote tumor progression. This speculation aligns with the results of subsequent transcriptome analyses [87].

Furthermore, experiments have revealed that inhibiting YAP/TAZ impedes the polarization of M2-like TAM, suggesting that the YAP/TAZ pathway plays a certain role in mediating macrophage M2 polarization induced by Nogo-B overexpression in the HCC microenvironment [90–92]. However, the precise mechanism by which YAP/TAZ promotes M2 polarization in TAM remains elusive. Knowing that YAP/TAZ is involved in lipid metabolic reprogramming, it is plausible that its hyperactivation, triggered by heightened Nogo-B signaling, leads to excessive accumulation and release of FA through increased activation of SREBP1, which may furnish HCC cells with an ample energy source, thereby promoting HCC progression and fostering the creation of an immunosuppressive TME, ultimately driving the polarization of TAM toward an M2 phenotype. Further elucidation of this mechanism is warranted.

While our attention is primarily directed toward understanding the reprogramming of TAM lipid metabolism via SREBP in HCC, it is crucial to recognize the significance of its upstream pathways, such as the mTOR kinase. mTOR kinase serves as a critical regulator of protein synthesis, bridging nutrient sensing with cell growth and cancer progression, and it participates in the modulation of various metabolic pathways including gluconeogenesis, glutamine metabolism, and lipid metabolism [71, 93]. Moreover, the substrates for the FA de novo synthesis pathway can originate from different precursors of the tricarboxylic acid (TCA) cycle, glycolysis, and the pentose phosphate pathway (PPP) [94]. It is therefore imperative to consider the impact of the upstream SREBP pathway on other energy metabolic pathways associated with lipid synthesis in investigating the specific pathways that directly govern lipid synthesis.

Indeed, certain experiments have indicated that restraining lipid de novo synthesis through the inhibition of the mTOR-mediated regulation of the SREBP pathway holds promise for preventing HCC [95]. Therefore, elucidation of the precise mechanism underlying this inhibition should be an attractive avenue for the development of related pharmaceutical agents.

It is worth noting that previous studies have reported a loss of LKB1 protein expression in 41% osteosarcoma patients, most presenting as mTORC1 activation [96]. In contrast, under the condition of nutrient deficiency, normal macrophages activate AMPK, which increases FA catabolism but inhibits mTOR activity [97]. Hence, there may be a correlation between the mechanism of mTOR in lipid regulation and the regulation of the LKB1-AMPK pathway. Further investigation on both pathways is required to clarify their associations with TAM lipid metabolism reprogramming for the sake of yielding new therapeutic insights (Supplementary Fig. 3).

#### Lysophosphatidic acid

Lysophosphatidic acid (LPA) is a bioactive phospholipid with diverse roles by exerting its effects through binding to the LPA receptor (LPAR) [98]. In certain cancer types, increased LPAR expression was found to be associated with heightened LPA signaling [99]. Autotaxin (ATX) is implicated in the increase in extracellular LPA production. Currently, it has been discovered that ATX and LPA signaling pathways are involved together in the progression of chronic inflammatory diseases, fibrosis, and cancer. In addition, relevant studies have demonstrated that disrupting ATX in hepatic cells leads to lipid homeostasis imbalance, which can mitigate the progression of HCC. Therefore, metabolic regulation of ATX also holds significance in the progression of HCC [98, 100].

LPA is implicated in various pathways and contributes to tumor progression and modulation of the TME. A pertinent previous investigation linking macrophage metabolic regulation to the ATX/LPA/LPAR axis explored its potential as a therapeutic target for neuropathic pain, finding that activation of macrophages/microglia led to LPA level elevation and initiated a self-perpetuating cycle of de novo LPA synthesis. This activation process promoted inflammation-mediated axonal demyelination, ultimately culminating in neuropathic pain [101].

The Agpat4/LPA/p38/p65 axis emerges as a promising therapeutic target in colon cancer. Research indicates that dysregulated elevation of 1-acylglycerol-3-phosphate O-acyltransferase 4 (Agpat4) attenuates the pro-inflammatory effects of LPA by suppressing the expression of pro-inflammatory factors (such as IL-1β, IL-6, and TNFα) in macrophages. Notably, flow cytometry in a previous study demonstrated that the infiltration of M1-like TAM was reduced in tissues from colon cancer patients who exhibited high Agpat4 expression [102].

Additionally, high LPA expression may affect macrophage development by activating the AKT/mTOR signaling pathway [103]. Although whether LPA regulation by other cell types or by macrophages themselves impacts the progression of HCC and TME remains unclear, previous findings in other cancers suggest that LPA-mediated metabolic regulation could contribute to a pro-inflammatory milieu in the TME. This could potentially promote the formation of M1-like TAMs by exerting an anti-tumor effect and impeding the progression of HCC. Further investigation is warranted to elucidate this process and its specific underlying mechanisms.

In summary, the regulation of metabolic synthesis of various lipids in macrophages may play a role in modulating HCC progression, and increased synthesis of different lipids does not always correlate positively with accelerated cancer progression. For instance, heightened cholesterol synthesis may contribute to the acceleration of HCC progression by promoting the destruction of normal cells. Conversely, elevated LPA synthesis may foster the creation of a pro-inflammatory environment in the TME, thereby inhibiting the progression of HCC. However, it is crucial to contextualize the regulation of FA synthesis. For example, TAMs are maintained intracellularly in a high-lipid environment, but the source of lipids is not primarily from the individual synthetic pathways themselves. Studies indicate that M1-like TAMs exhibit significant FA de novo synthesis activity, which serves various functions such as providing energy supply, facilitating prostaglandin biosynthesis, and inducing post-translational modifications to regulate pro-inflammatory macrophage responses, ultimately enhancing tumor cell killing. On the other hand, the lipid source of M2-like TAMs is primarily involved in CD36-mediated uptake and lysosomal acid lipase (LAL)-mediated lysosomal lipolytic uptake [104]. Given the functional variability between M1 and M2-like TAMs, it can be speculated that HCC progression may be inhibited when TAMs predominantly utilize the lipid synthesis pathway, whereas promotion may occur in the opposite scenario.

### Lipid oxidative decomposition of TAM

Lipid oxidative decomposition represents a critical energy source within the TME, which is primarily driven by TG catabolism and FAO. Notably, previous animal experiments have demonstrated that within the TME, gene expression patterns associated with FA β-oxidation and FAO-related genes are markedly upregulated in TAMs compared to control macrophages (MΦs), suggesting that TAMs with high-lipid accumulation primarily derive their energy through elevated levels of FAO rather than glycolysis [23]. However, it is important to recognize that both M1-like and M2-like TAMs undergo distinct metabolic reprogramming processes concerning lipid oxidative catabolism.

#### Differential reprogramming of lipid oxidative catabolism in M1/M2-like macrophages in HCC

The predominance of M2-like TAM in the TME typically fosters HCC progression, while the M1 phenotype tends to exhibit pro-inflammatory properties and inhibit HCC advancement. In the advanced stage of the HCC microenvironment, M2 macrophages constitute a significant proportion. Analysis through the lens of metabolic reprogramming shows that TAM polarization toward the M1 phenotype is primarily metabolically regulated by aerobic glycolysis, akin to the Warburg effect observed in HCC cells. In contrast, the transition of TAM to the M2 phenotype relies on the elevated lipid milieu established by HCC cells within the TME, leading to metabolic reprogramming characterized by increased FA uptake and oxidation [22, 105], [106]. Evidence indicates that Silent Information Regulator 4 (SIRT4) is significantly downregulated in HCC and promotes M2 polarization of macrophages through the FAO-PPARδ-STAT3 axis [107]. However, it is crucial to note that heightened FA uptake and oxidation are linked to M2-like TAM, but this may also result from lipid metabolic effects stemming from the recruitment of effector T cells and NK cells prompted by pro-inflammatory factors secreted by other macrophage subsets such as CXCL10, IL-1β, and IL-10. Therefore, further investigation into how M2-like TAM utilizes FA within the TME is warranted [106]. Inhibiting M2-like TAM formation by targeting this process may represent an effective therapeutic strategy for future HCC treatment.

Upon polarization, pro-inflammatory signals have been observed to induce the M1 phenotype in macrophages, which typically increases the storage of LD in M1 macrophages due to heightened synthesis of TG and FA, along with diminished lipid metabolism marked by decreased FAO. This alteration is significant in regulating M1 phagocytosis, for LD serves as the site for energy storage, sequester toxic lipids, and mitigate excessive endoplasmic reticulum (ER) stress [108]. Additionally, in M2-like TAMs, FA primarily originates from TGs, which are taken up via CD36 and subsequently hydrolyzed by LAL. This process is believed to activate M2-like TAMs by bolstering oxidative phosphorylation, thus enhancing the spare respiratory capacity (SRC), prolonging cellular lifespan, and augmenting gene expression [109]. The specific mechanisms by which both types of TAMs regulate lipid oxidation metabolism in HCC still require further investigation. This may potentially become one of the targets in the future for inducing anti-cancer phenotypes in macrophage populations through lipid metabolism regulation.

#### Mechanism of M1-type polarization

The mechanisms underlying M1-like polarization in HCC, particularly concerning lipid oxidative catabolism, remain largely unexplored. However, the existing literature suggests that M1 polarization predominantly activates the glycolysis pathway to rapidly meet the heightened demand for bactericidal activity, while M2 polarization favors a more efficient and sustained energy metabolism pathway, such as FA oxidation. Indeed, studies have demonstrated that blocking oxidative metabolism not only selectively impedes the differentiation process of M2-type macrophages but also enhances the proliferation and expression of M1-like macrophages [110]. Therefore, it is reasonable to speculate that M1-like polarization may be achieved by downregulating FA oxidation or TG catabolism, and this process could potentially be inhibited during the progression of HCC.

However, it should be noted that in melanoma models, macrophage CD40 signaling was found to promote the re-education of TAM toward an anti-tumor phenotype while concurrently enhancing FAO and glutamine metabolism [111]. This finding of the study underscores the notion that the effect of lipid metabolic reprogramming on TAM polarization in HCC may be bidirectional.

#### Mechanism of M2-type polarization

In recent years, significant progress has been made in studying the mechanisms related to the reprogramming of TAMs toward M2-type polarization through lipid oxidative catabolism. A pivotal aspect of this process involves investigating targets within the lipid oxidative catabolic pathway. Previous research has demonstrated that inhibiting FAO can prevent M2-type polarization. Carnitine palmitoyl transferase (CPT) serves as the rate-limiting enzyme in FAO. Pharmacological targeting of the CPT system has been employed in these investigations, aiming to impede FA translocation within the mitochondria and consequently inhibit FAO, thereby suppressing the M2-type polarization of TAMs [112]. This line of research has also paved the way for exploring additional targets associated with lipid oxidative metabolism.

IL-25, as mentioned previously in the context of regulating TAM lipid uptake, has been shown in related studies to increase the expression of β-oxidase and stimulate lipolysis by alternatively activating macrophages, thereby promoting M2 macrophage polarization [26]. This suggests that IL-25 may contribute to high levels of FAO in M2 macrophages. Consequently, exploring strategies to impede the formation of an immunosuppressive environment by inhibiting IL-25 could hold potential for investigating approaches to slow down the progression of HCC.

IL-1β, a manifestation of IL-1, is primarily produced by monocytes and macrophages and plays a crucial role in cellular defense, tissue repair, pain, inflammation, and autoimmunity across various tissues. Studies have indicated that M2 macrophages promote the secretion of IL-1β in a FAO-dependent manner, which in turn enhances HCC cell proliferation, migration, and invasion. Additionally, IL-1β induction has been found to rely on reactive oxygen species (ROS) and NLRP3 [113]. Hence, NLRP3 may not only regulate lipid storage in TAMs but also be closely associated with TAM lipid oxidation in HCC.

Receptor-interacting protein kinase 3 (RIPK3), a central necrosis factor, is implicated in the metabolic reprogramming of HCC. Studies have revealed that in HCC, RIPK3 is downregulated in TAMs, leading to enhanced FA metabolism by reducing ROS production and inhibiting caspase-1-mediated peroxisome proliferator-activated receptor (PPAR) cleavage, thereby promoting PPAR activation and M2 polarization in the TME. Conversely, experiments have demonstrated that upregulation of RIPK3 or blockade of FAO can reverse the immunosuppressive activity of TAMs and inhibit the progression of HCC. Therefore, targeted molecular therapy aimed at RIPK3 may represent a promising approach for regulating immune metabolism in HCC [14].

Trigger receptor 2 (TREM2), expressed in bone marrow cells, is an innate immune receptor found on the surface of myeloid cells, including monocytes, macrophages, osteoblasts, and microglia [114]. Animal experiments have demonstrated that mice lacking TREM2 exhibit exacerbated liver injury and inflammation in the early stages of HCC, suggesting a protective role for TREM2 during early HCC progression [115, 116]. However, analysis of single-cell transcriptomes from human HCC tissues has revealed that TREM2 is primarily expressed by TAMs, akin to lipid-associated macrophages (LAMs). Moreover, tumors with high TREM2 expression tend to have a higher proportion of M2-like TAMs, which, along with TREM2 + LAM-like cells, exert synergistic immunosuppressive effects to drive HCC progression. Pathway energy analysis suggests that TREM2 + macrophage subtypes exhibit a higher expression fraction in glutamine metabolism and lipolysis. Glutamine metabolism generates α-ketoglutarate, which is crucial for promoting FAO and mitochondrial oxidative phosphorylation (OXPHOS), thereby driving M2 polarization of macrophages [104, 117]. Consequently, excessive lipid metabolism may also help promote immunosuppression in HCC.

The targets involved in lipid metabolism reprogramming in TAMs are summarized in Tables 1 and 2. Although the aforementioned targets primarily focus on inducing M2 polarization of TAMs through reprogramming of lipid degradation metabolism in HCC, it is crucial to further investigate M1-type polarization. However, it is important to note that in the analysis of TAM lipid oxidative catabolic reprogramming, a complete separation between lipid oxidative catabolism and uptake and synthesis may not exist. Malonyl coenzyme A, the primary substrate for FA synthesis, can inhibit FAO by blocking carnitine palmitoyl transferase 1 (CPT-1), which inhibits FA transport to mitochondria [118]. Sterol regulatory element-binding protein 1 (SREBP1), another central regulator of FA metabolism downstream of LKB1-AMPK signaling, also regulates FA, TG, and cholesterol synthesis by controlling the transcription of ATP citrate lyase (ACYL), acetyl-CoA carboxylase (ACC), stearoyl-coenzyme A desaturase 1 (SCD1), and FA synthase (FASN) [81, 119]. Additionally, high expression of IL-25 in HCC has been shown to increase TAM lipid uptake and enhance oxidative lipid catabolism by alternately activating TAM, leading to increased expression of lipolytic enzymes and β-oxidase [26]. Hence, exploring the interactions between various pathways involved in TAM lipid metabolism reprogramming and their impacts on TAMs themselves and the HCC process represents a potential direction of future research.

**Table 1:** Target for lipid metabolism reprogramming in TAM

Target	Major effects of lipid metabolism in TAM	References
CD36	Increases lipid intake	[23]
IL-25	Enhances absorption of lipids	[26, 27]
	Increases β-oxidase expression and lipolysis by alternatively activating macrophages	[26]
FABP	Regulates lipid uptake and storage	[41]
LKB1	Activation of the LKB1-AMPK signaling pathway inhibits de novo FA synthesis	[63, 64]
Ch25h	Upregulated Ch25h maintains inhibitory effects on SREBP2 activation and cholesterol synthesis	[83]
SCAP	Increases cholesterol accumulation when it mediates the activation of SREBP-1a	[84]
Mst1, Mst2	Inhibits YAP/TAZ transcriptional activity to inhibit de novo lipid production	[87, 88]
LAL	Mediates lysosomal lipolysis uptake	[104]

**Table 2:** Potential target for lipid metabolism reprogramming in TAM

Target	Major effects	References
miR-122	Decreases TG accumulation in the liver and peripheral tissues	[48, 120]
LKB1	Low LKB1 activation levels of macrophages in the cardiovascular system promote lipid uptake and foam cell formation	[43–45]
SREBP	Key nuclear transcription factors that regulate lipid metabolism	[69]
Nogo-B	High expression of Nogo-B can activate the YAP/TAZ pathway and mediate the M2 polarization process of macrophages	[92]
LPA	High expression of the Agpat4/LPA/p38/p65 axis showed less invasion of M1-like TAM in colon cancer microenvironment	[102]
NLRP3	Increases IL-1β to enhance proliferation, migration, and invasion of HCC cells	[113]
TREM2	TREM2 + macrophages have higher levels of glutamine metabolism and lipolysis	[104, 117]
Proinflammatory factor (e.g., CXCL10, IL-1β, and IL-10)	Possibly associated with increased FA uptake and oxidation of TAM	[101]
CPT	Rate-limiting enzymes in FAO	[14]
RIPK3	RIPK3 is down-regulated in TAM to promote FA metabolism	[14]
CD40	Promotes FAO	[111]

## Study on HCC therapy targeting TAM

While there are currently no ongoing drug trials specifically targeting lipid metabolic reprogramming in TAMs for HCC, it is worth considering relevant studies targeting TAMs for the treatment of HCC.

The CCL2-CCR2 axis mediates monocyte and TAM chemotaxis within the TME. Experimental evidence has demonstrated the involvement of the CCL2-CCR2 axis in TAM accumulation in esophageal cancer [18]. Furthermore, clinical studies have indicated that CCR2 receptor antagonists can inhibit the recruitment of inflammatory monocytes, TAM infiltration, and M2 polarization in HCC, suggesting a potential therapeutic strategy for treating HCC [121].

CSF-1, a cytokine, plays a crucial role in regulating differentiation and function of macrophages and is implicated in TAM infiltration in HCC [122]. As previously discussed, CSF-1 can promote M2 macrophage polarization and the establishment of an immunosuppressive microenvironment. Studies have indicated that blocking the CSF-1 receptor (CSF-1R) with inhibitors like PLX3397 slows down HCC growth by modulating macrophage polarization rather than depleting TAM altogether [122].

In addition to its association with M1 macrophages and inflammatory properties, IL-6 also plays a role in promoting the expansion of cancer stem cells within the HCC microenvironment [123]. Studies have demonstrated that tocilizumab, an IL-6 receptor inhibitor, can effectively block the pro-tumorigenic effects mediated by TAMs in HCC progression [123]. However, further investigation is required to clarify the paradoxical role of IL-6 secreted by M1 macrophages with tumor-killing activity in promoting HCC progression.

Certainly, there are ongoing studies targeting TAM lipid metabolism reprogramming for the treatment of other tumors. In EGFR-driven lung adenocarcinoma (LUAD), tumor-associated alveolar macrophages (TA-AMs) can reduce EGFR phosphorylation and inhibit LUAD progression by enhancing GM-CSF-PPARγ signaling and suppressing PPARγ-driven signaling to inhibit cholesterol flux toward tumor cells. Furthermore, treatment with statins and concurrent blockade of PPARγ in TA-AMs can suppress tumor progression and enhance pro-inflammatory immune responses [124].

In addition to the targets mentioned above, numerous ongoing studies are exploring various strategies to target TAMs for the treatment of HCC. Many targets discussed in this paper, particularly those related to the reprogramming of TAM lipid metabolism, hold significant promise as potential breakthroughs in the future treatment of HCC.

## Conclusion and future perspectives

In this review article, we have endeavored to consolidate the potential implications of TAMs in HCC progression through metabolic reprogramming by laying a specific focus on lipid metabolism. We have not only tried to highlight the roles of both M1 and M2 macrophage fractions in TAMs within the context of HCC progression and elucidate their distinct molecular mechanisms in various stages of lipid metabolism but also make a comprehensive summary of TAM polarization influenced by lipid metabolism reprogramming. The details are shown in Supplementary Table 3.

Given the limited number of studies directly investigating the correlation between HCC and TAM lipid metabolism reprogramming, we have extrapolated insights from the broader literature on TAMs and lipid metabolism in other tumor types. Through reasonable speculation based on these relationships, we have outlined the characteristics and mechanisms of TAM lipid metabolism reprogramming in the context of HCC progression.

Several identified targets, such as SREBP and LKB1, exert their effects across multiple lipid metabolic pathways or stages, including storage, uptake, and synthesis. It is our hope that by delineating these targets and pathways, we can shed light on the potential avenues for the prevention, treatment, and prognostic assessment of HCC. Furthermore, these findings may provide valuable directions for future investigations into the effects of lipid metabolic reprogramming on HCC.

In the context of HCC, macrophage targeting has garnered considerable attention, particularly due to resistance of the disease to immune checkpoint therapies, which is closely associated with the abundant presence of TAMs in the HCC microenvironment and their M2 phenotype-mediated immunosuppression. To address this, various immunotherapeutic strategies have been explored to target TAMs. These strategies primarily aim to:

- Inhibit monocyte/macrophage recruitment: By targeting chemokines and their receptors involved in monocyte and macrophage recruitment, such as the CCL2-CCR2 axis, efforts are made to reduce the influx of monocytes into the TME, thereby limiting TAM accumulation.
- Deplete TAMs: Approaches involving the selective depletion of TAMs within the TME have been investigated. This could involve targeting specific surface markers or signaling pathways crucial for TAM survival and function.
- Manipulate TAM differentiation: Efforts have been made to develop strategies that can modulate TAM polarization toward desired phenotypes, particularly shifting from the immunosuppressive M2 phenotype toward the pro-inflammatory M1 phenotype. This could involve targeting signaling pathways or cytokines involved in TAM polarization.
- Utilize TAM-mediated drug delivery: TAMs can serve as drug delivery vehicles within the TME. By leveraging their phagocytic capacity, drug-loaded nanoparticles or other therapeutic agents can be targeted specifically to TAMs, thus allowing for localized delivery to the tumor site [125].

Overall, targeting TAMs holds promise as a therapeutic approach for HCC treatment, and ongoing research efforts are focused on refining these strategies to improve their efficacy and minimize off-target effects.

Current studies have highlighted the significant role of chemokines such as CCL2 and CCL5 in promoting tumor infiltration and polarization of TAMs toward the pro-oncogenic M2 phenotype. In response, a single-domain antibody named BisCCL2/5i has been developed to specifically bind and neutralize CCL2 and CCL5, showing promising results in mouse models of HCC. It is speculated that combining BisCCL2/5i with PD-L1 inhibitors could potentially improve survival outcomes in human HCC patients [126]. However, it is important to note that macrophages are crucial for the efficacy of chemotherapy and immunotherapy [127]. Complete depletion of TAMs from the TME may not be the most effective treatment strategy. Instead, targeting lipid metabolic reprogramming to promote the transformation of TAMs from the M2 to M1 phenotype may hold more promise. Currently, efforts in this direction primarily focus on reprogramming TAM differentiation through coordinating their phagocytic function, adjusting T cell anti-tumor activity, and targeting complement-related receptors. For instance, inhibiting the CD47-SIRPα pathway enhances macrophage phagocytosis of tumor cells, activation of CD40 promotes TAM reprogramming toward an anti-tumor phenotype and enhances the accumulation and longevity of T cell receptor-engineered cells. Additionally, inhibitors of C3a and C5a receptors can reduce the recruitment and activation of immunosuppressive cell subpopulations [125]. Despite these promising avenues, there are currently no ongoing drug trials or clinical studies specifically targeting lipid metabolic reprogramming for HCC. However, continued research in this area holds potential for the development of novel therapeutic approaches for HCC treatment.

The intricate and unique metabolic pathways of various cells grant them a decisive advantage in nutrient competition, influencing processes such as cell division, differentiation, and apoptosis, which directly impact cancer progression. Consequently, targeting metabolic pathways that indirectly influence cancer processes has garnered significant interest. The concept of cell-type-specific metabolic pathway inhibition has emerged as a promising strategy in anti-cancer immunotherapy [128]. Indeed, while glucose metabolic reprogramming, such as the Warburg effect, has been extensively investigated in cancer metabolism, targeting this phenomenon has become a prominent anti-cancer strategy [129]. However, recognizing the critical role of lipids in the progression of various cancers, there is a growing need to delve deeper into cancer lipid metabolic reprogramming. Consequently, inhibiting cancer progression by influencing the metabolic reprogramming of TAMs holds promise as a highlight in HCC treatment.

Indeed, navigating the complexities of lipid metabolic reprogramming in the context of TAM polarization poses significant challenges. The multifaceted effects of altering lipid metabolism on TAM polarization underscore the need for a nuanced understanding of the specific mechanisms involved at various stages of HCC development. Moreover, therapeutic interventions targeting TAM lipid metabolism should address potential off-target effects on other cells and tissues, emphasizing the importance of precision medicine approaches [130]. Future studies on targeted TAM lipid metabolism therapies should prioritize elucidating the primary direction and precise mechanisms by which lipid metabolic reprogramming impacts TAM polarization in HCC. Advancements in technology, such as the development of nanoparticles, have offered promising avenues for enhancing the precision of treatment targeting TAM in the liver [131]. However, further exploration through well-designed experiments is essential to refine the details of these therapeutic strategies.

Lastly, it is important to note that while investigating the impact of TAM lipid metabolic reprogramming on the HCC process, it is crucial to consider the reciprocal influence of HCC on TAMs. Recent studies have documented that lipid accumulation within HCC cells can induce immunosuppressive alterations in co-cultured macrophages. Specifically, the steatogenic HCC microenvironment is characterized by infiltration of a substantial number of M2 macrophages, accompanied by upregulation of cytokines and chemokines that promote M2 polarization and immunosuppression [132]. Moreover, research has demonstrated that palmitic acid (PA) accumulation in HCC cells may lead to the upregulation of programmed death-ligand 1 (PD-L1) expression and increased expression of colony-stimulating factor 1 (CSF1), C-X-C motif chemokine ligand 8 (CXCL8), and transforming growth factor-beta 1 (TGF-β1) at both mRNA and secreted protein levels. Concurrently, elevated levels of CD206 and IL-10 were observed in co-cultured macrophages, which directly and indirectly promoted M2 macrophage polarization and the establishment of an immunosuppressive environment [132]. Hence, it is imperative to explore whether lipid changes within HCC cells affect macrophage polarization through TAM lipid metabolic reprogramming. This line of inquiry holds promise for uncovering the intricate interplay between lipid metabolism and immune regulation within the HCC microenvironment.

### Supplementary Information

Below is the link to the electronic supplementary material.

Supplementary file1 (DOCX 147 kb)

## Data Availability

Not applicable.
