# Supplementary material for: The significance of lipid metabolism reprogramming of tumor-associated macrophages in hepatocellular carcinoma
Source: Cancer Immunol Immunother. 2024 Jul 2;73(9):171. doi: 10.1007/s00262-024-03748-9 (PMC11220057; doi:10.1007/s00262-024-03748-9)
Supplement: Supplementary file 1 — Supplementary file1 (DOCX 147 kb) [file 262_2024_3748_MOESM1_ESM.docx]

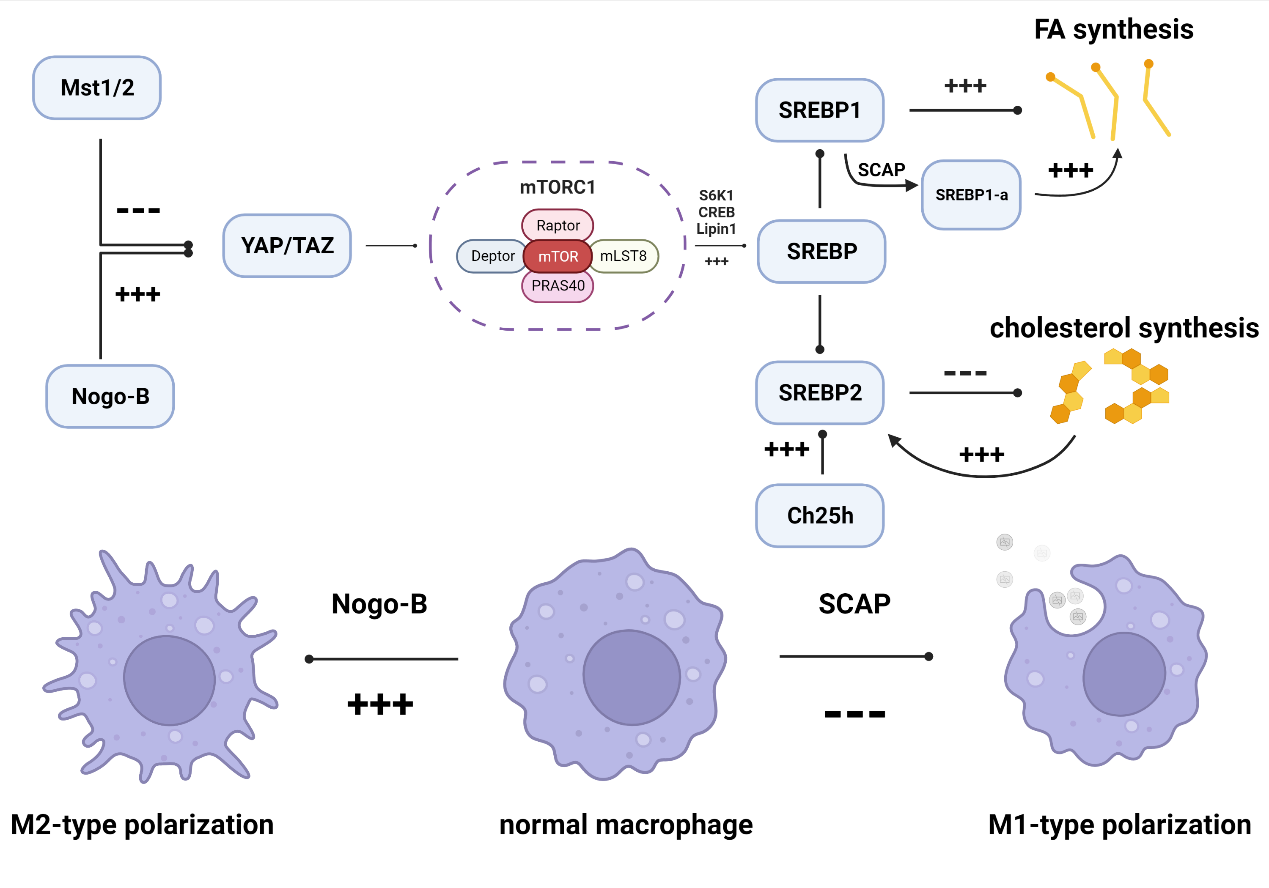


**Figure 3**. SREBP with Lipid Synthesis Reprogramming can be summarized as follows: 1) Nogo-B can activate YAP/TAZ, thereby promoting the polarization of M2 macrophages. 2) Mst1/2 kinases can inhibit the activity of YAP/TAZ. 3) YAP/TAZ promotes the synthesis of SREBP through mTORC1 via proteins such as S6K1, CREB, and Lipin1. 4) SREBP is classified into two isoforms: SREBP1 and SREBP2. SREBP1, particularly SREBP1-a, promotes fatty acid synthesis through SCAP, while SREBP2 inhibits cholesterol synthesis. However, the synthesis of SREBP2 can be promoted conversely. 5) Ch25h promotes the synthesis of SREBP2. 6) SCAP inhibits the polarization of M1. Source Created with BioRender.com.

**Table 3.** Effect of lipid metabolic reprogramming on TAM polarization.

| Target | Effect |
| --- | --- |
| Increased FA uptake  Decreased FA uptake  Increased TG uptake  Decreased TG uptake  Increased cholesterol efflux  Decreased cholesterol accumulation  Increased FA de novo synthesis  Increased LPA synthesis  Increased lipid raft consumption  Increased FAO  Decreased FAO  Decreased TG decomposition | M2 polarization  M1 polarization  M2 polarization  M1 polarization  M2 polarization  M1 polarization  M1 polarization  M1 polarization  M2 polarization  M2 polarization  M1 polarization  M1 polarization |
